# Influence of exposure conditions on helium transport and bubble growth in tungsten

**DOI:** 10.1038/s41598-021-93542-9

**Published:** 2021-07-19

**Authors:** Rémi Delaporte-Mathurin, Mykola Ialovega, Etienne A. Hodille, Jonathan Mougenot, Yann Charles, Elodie Bernard, Céline Martin, Christian Grisolia

**Affiliations:** 1 CEA, IRFM, 13108 Saint-Paul-lez-Durance, France; 2 Laboratoire des Sciences des Procédés et des Matériaux, LSPM, CNRS, Université Sorbonne Paris Nord, UPR 3407, -93430 Villetaneuse, France; 3 CNRS, PIIM, Aix Marseille University, 13397 Marseille, France

**Keywords:** Mathematics and computing, Applied mathematics, Materials science, Theory and computation

## Abstract

Helium diffusion, clustering and bubble nucleation and growth is modelled using the finite element method. The existing model from Faney *et al.* (Model Simul Mater Sci Eng 22:065010, 2018; Nucl Fusion 55:013014, 2015) is implemented with FEniCS and simplified in order to greatly reduce the number of equations. A parametric study is performed to investigate the influence of exposure conditions on helium inventory, bubbles density and size. Temperature is varied from 120 K to 1200 K and the implanted flux of 100 eV He is varied from $10^{17}\,{\text{m}^{-2}\, \text{s}^{-1}}$ to $5 \times 10^{21}\, {\text{m}^{-2}\, \text{s}^{-1}}$. Bubble mean size increases as a power law of time whereas the bubble density reaches a maximum. The maximum He content in bubbles was approximately $4 \times 10^{8}$ He at $5 \times 10^{21}\,{\text{m}^{-2}\, \text{s}^{-1}}$. After 1 h of exposure, the helium inventory varies from $5 \times 10^{16} \,{\text{m}^{-2}}$ at low flux and high temperature to $10^{25} \,{\text{m}^{-2}}$ at high flux and low temperature. The bubbles inventory varies from $5 \times 10^{12}$ bubbles m$^{-2}$ to $2 \times 10^{19}$ bubbles m$^{-2}$. Comparison with experimental measurements is performed. The bubble density simulated by the model is in quantitative agreement with experiments.

## Introduction

In fusion devices, extreme fluxes of helium (He) and hydrogen (H) are expected. These fluxes will be mostly located on the tungsten (W) divertor which will also exhaust the plasma He ashes.

**Figure 1:**
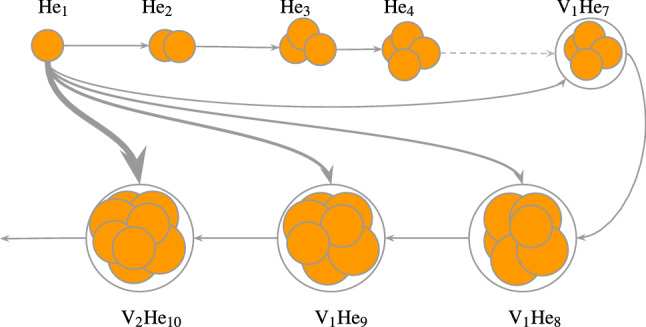
Representation of He clustering in solids. Dissociation is omitted for simplification purposes. The grey arrows thicknesses represent the magnitude of the reaction rate between mobile He$_1$ and other clusters at the same distance.

Due to a strong W-He repulsion, interstitial He in W tend to react and form clusters^1,2^ (see Fig. 1). Eventually, when mobile clusters reach a critical size, trap-mutation (also called self-trapping) will occur. Frenkel pairs (self-interstitial W atom and vacancy) will be produced and the mobile clusters will sit in the created vacancies^3^. At this point, the clusters are immobile and will continue to grow by absorbing small helium clusters and act as nuclei for bubble formations. When the cluster is over-pressurised, additional Frenkel pairs will be created. He bubbles can then form in W and their morphology depends on the exposure conditions^4–7^. Such bubbles have been observed using Molecular Dynamics (MD)^3,8–12^ and Object Kinetic Monte Carlo^13,14^. He bubbles can alter the mechanical properties of W^6,15,16^ and reduce the thermal properties of components^17,18^. Eventually, when over-pressurised bubbles are close to the surface, bursting can occur as shown by Sefta *et al.* in MD simulations^12,19^. Bursting greatly modifies surface morphologies by increasing the roughness and producing craters^4^ and W-fuzz^7,20–23^. Moreover, He exposure can alter H retention in W^4,24–28^.

An effort has been made to assess He transport in W using atomistically informed macroscopic models called cluster dynamics models implemented using finite differences. Some examples of such implementations are the work of Faney *et al.*^29,30^ and the Xolotl code^31^. The simulated results are promising but these codes require substantial computational resources considering the thousands of equations that need to be solved. The current study therefore proposes an approach to further simplify these models so that they can be easily implemented in finite element codes and later coupled to H transport modelling codes such as FESTIM^32^. This could serve as a base for H-He coupled simulations to better assess H transport in plasma facing materials and couple with additional physics like heat transfer.

The simplified model presented in this work is applied on a simple case and compared with existing results of the literature to ensure the model is not over-simplified. Then, the influence of exposure conditions is investigated by running a parametric study varying temperature and implanted particle flux. The results of this parametric study are analysed using a regression method previously employed^33^. Experiments are then conducted to quantitatively assess the He bubble density and size in He irradiated W. The current model is finally compared to these experimental results.

## Methodology

This section describes the He transport model and the grouped approach employed to simplify it.

### Helium clustering model

This model describes the evolution of the concentrations of pure interstitial He clusters (He$_x$) and mixed He-vacancies clusters (He$_x$V$_y$) that are formed by trap-mutation events.

The spatio-temporal evolution of each species of size *i* is defined by:

1 $$\begin{aligned} \frac{\partial c_i}{\partial t} = \nabla \cdot (D_i\nabla c_i) + \Gamma _i + R_i \end{aligned}$$

In Equation 1, the first term of the right hand-side is the diffusion term where ${D=D_0 \cdot \exp (-E_{\mathrm{diff}}/ (k_B \cdot T ) )}$ is the thermally activated diffusion coefficient expressed in ${\text{m}^2\, \text{s}^{-1}}$ with $E_{\mathrm{diff}}$ the diffusion activation energy in eV, $k_B$ the Boltzmann constant in eV K$^{-1}$ and *T* the temperature in K. If a species *i* is assumed to be immobile, its diffusion coefficient $D_i$ is zero. $\Gamma _i$ is the external production rate of species *i*.

The term $R_i$ is the coupling term due to reactions between species. A simple reaction between two species can be described as:

2 $$\begin{aligned} A + B {\rightleftharpoons }^{k^+_{\mathrm{A,B}}}_{k^-_{\mathrm{A,B}}} AB \end{aligned}$$

The forward rate constant $k^+_{A,B}$ is the clustering rate and is calculated using the theory of diffusion-limited reactions^34^:

3 $$\begin{aligned} k^+_{\mathrm{A,B}} = 4 \pi (r_{\mathrm{A}} + r_{\mathrm{B}}) (D_{\mathrm{A}} + D_{\mathrm{B}}) \end{aligned}$$

where $r_{\mathrm{A}}$ and $r_{\mathrm{B}}$ are the capture radii and $D_{\mathrm{A}}$ and $D_{\mathrm{B}}$ are the diffusion coefficients of species A and B respectively. The backward rate constant $k^-_{\mathrm{A,B}}$ is the dissociation rate and is obtained using chemical equilibrium principles^34^:

4 $$\begin{aligned} k^-_{\mathrm{A,B}} =\rho k^+_{\mathrm{A,B}}e^{\frac{-E_b}{k_B T}} \end{aligned}$$

where $\rho$ is the atomic density in ${\mathrm{m}^{-3}}$ ($\rho = {6.3 \times 10^{28}}\,{\text{m}^{-3}}$ for W), $k_B$ is the Boltzmann constant in eV K$^{-1}$, *T* is the temperature in K and $E_b$ is the binding energy for the reaction AB $\rightarrow$ A + B in eV.

The reaction term $R_i$ is the coupling term between concentrations and is expressed as:

5 $$\begin{aligned} R_i= \sum _{m} k^+_{m,i-m} c_m c_{i-m} - c_i \sum _m \left( k_{i, m}^+ c_{m} + k_{i+1}^- c_{i+1} - k_i^- c_i \right) \end{aligned}$$

In Equation 5, $c_i$ is the concentration of clusters of size *i* in m$^{-3}$. The first term corresponds to the reactions producing clusters of size *i*. The second one corresponds to the ones reacting with clusters of size *i*. The third term accounts for bigger clusters dissociating. Finally, the last term corresponds to clusters of size *i* dissociating.

### Grouped approach

Extending this clustering model to clusters containing millions of helium extremely increases the computational cost. A grouped approach proposed by Faney et al.^29^ for reducing the number of equations will therefore be employed. This technique consists in grouping the big clusters that have a similar behaviour in a single equation while explicitly accounting for smaller clusters.

The clustering equations can be written as follows:

6a $$\begin{aligned}&\frac{\partial c_1}{\partial t} = \nabla \cdot (D_1 \nabla c_1) + \Gamma + \sum _{i=2}^N k_{i}^- c_i - 2k_{1, 1}^+ c_1^2 - \sum _{i=2}^N k_{1,i}^+ c_1 c_i - \sum _{i=N+1}^\infty k_{1,i}^+ c_1 c_i \end{aligned}$$

6b $$\begin{aligned}&\frac{\partial c_2}{\partial t} = \nabla \cdot (D_2 \nabla c_2) - k_{1, 2}^+ c_1 c_2 + k_{1, 1}^+ c_1^2 - k_{2}^- c_2 + k_{3}^- c_3 \end{aligned}$$

6c $$\begin{aligned}&\vdots \nonumber \\&\frac{\partial c_i}{\partial t} = - k_{1, i}^+ c_1 c_i + k_{1, i-1}^+ c_1 c_{i-1} - k_{i}^- c_i \end{aligned}$$

6d $$\begin{aligned}&\frac{\partial c_{i+1}}{\partial t} = - k_{1, i+1}^+ c_1 c_{i+1} + k_{1, i}^+ c_1 c_i \nonumber \\&\vdots \end{aligned}$$

where *N* is some threshold required for the grouping technique.

In order to simplify this model, the following quantities are defined:

7 $$\begin{aligned}&c_b = \sum _{i=N+1}^\infty c_i \quad \text{ : total concentration of clusters containing more than}\; N \text{ He} \end{aligned}$$

8 $$\begin{aligned}&\langle i_b \rangle = \frac{1}{c_b} \sum _{i=N+1}^\infty i c_i \quad \text{ : average He content in} \; c_b \end{aligned}$$

9 $$\begin{aligned}&\langle r_b \rangle = \frac{1}{c_b}\sum _{i=N+1}^\infty r_i c_i \quad \text{ : average radius in} \; c_b \end{aligned}$$

10 $$\begin{aligned}&\langle k_b^+ \rangle = \frac{1}{c_b}\sum _{i=N+1}^\infty k_{1,i}^+ c_i = 4 \pi D_1 (r_1 + \langle r_b \rangle ) \quad \text{ : average clustering rate in} \; c_b \end{aligned}$$

Clusters with more than *N* He ($c_b$) will be referred as “bubbles” in the following.

Equation 6 therefore reads:

11a $$\begin{aligned}&\frac{\partial c_1}{\partial t} = \nabla \cdot (D_1 \nabla c_1) + \Gamma + \sum _{i=2}^N k_{i}^- c_i- 2k_{1, 1}^+ c_1^2 - \sum _{i=2}^N k_{1,i}^+ c_1 c_i - \langle k_b^+ \rangle c_1 c_b \end{aligned}$$

11b $$\begin{aligned}&\frac{\partial c_2}{\partial t} = \nabla \cdot (D_2 \nabla c_2) - k_{1, 2}^+ c_1 c_2 + k_{1, 1}^+ c_1^2 - k_{2}^- c_2 + k_{3}^- c_3 \end{aligned}$$

11c $$\begin{aligned}&\vdots \nonumber \\&\frac{\partial c_N}{\partial t} = - k_{1, N}^+ c_1 c_N + k_{1, N-1}^+ c_1 c_{N-1} - k_{N}^- c_N \end{aligned}$$

11d $$\begin{aligned}&\frac{\partial c_b}{\partial t} = k_ {1,N}^+ c_1 c_N \end{aligned}$$

11e $$\begin{aligned}&\frac{\partial (\langle i_b \rangle c_b)}{\partial t} = (N+1)k_ {1,N}^+ c_1 c_N + \langle k_b^+ \rangle c_1 c_b \end{aligned}$$

The mean radius of pure He clusters^30^ is given by:

12 $$\begin{aligned} r_{\mathrm{He}_x} = r_{\mathrm{He}_1} + \left( \frac{3}{4\pi } \frac{a_0^3}{10} x \right) ^{1/3} - \left( \frac{3}{4\pi } \frac{a_0^3}{10} \right) ^{1/3} \end{aligned}$$

with $$r_{\mathrm{He}_1} = {0.3}\,\mathrm{nm}$$.

Several assumptions are made:

- The average radius is assumed to be a function of $\langle i_b \rangle$:
  13 $$\begin{aligned} \begin{aligned} \langle r_b \rangle&= r(\mathrm{He}_{\langle i_b \rangle }\mathrm{V}_{\langle i_b \rangle /4}) \\&= r_{\mathrm{He}_0 \mathrm{V}_1} + \left( \frac{3}{4 \pi } \frac{a_0^3}{2} \frac{\langle i_b \rangle }{4} \right) ^{1/3} - \left( \frac{3}{4 \pi } \frac{a_0^3}{2} \right) ^{1/3} \end{aligned} \end{aligned}$$
  with $a_0 = {0.318}\,\text{nm}$ the lattice parameter and $r_{\mathrm{He}_0 \mathrm{V}_1} = a_0 \sqrt{3}/4$. The average radius $\langle r_b \rangle$ is assumed to be only dependent on $\langle i_b \rangle$. The number of vacancies in bubbles is assumed to be $\langle i_b \rangle /4$. This assumption is motivated by MD computations showing that trap mutation events occur for every four additional helium in large vacancy-helium clusters. Moreover, theoretical models for He bubbles growth in metal suggest a similar trend^35^.
- Dissociation of large clusters is neglected (i.e. $k_i^- = 0$ for $i>N$). Indeed, the activation energy for trap mutation events is lower than that of He or vacancy emission^3^. Dissociation of large clusters by vacancy or He emission is therefore negligible.

**Figure 2:**
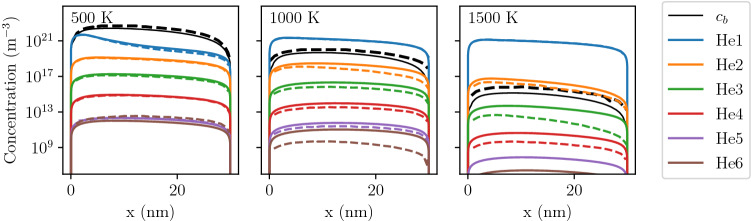
He clusters concentration profiles in the tendril at 500 K, 1000 K and 1500 K. Comparison between the current implementation (solid) and Faney’s results^30^ (dashed). Discrepancies at high temperature are likely due to the use of a different set of dissociation energies. At 1000 K and 1500 K the dashed and solid profiles of He$_1$ overlap.

The current implementation further simplifies Faney’s model^29^:

- Interactions with self-interstitial atoms or pre-existing vacancies are not taken into account. In this work, the only dissociations are He emissions from small mobile clusters and trap-mutation for large clusters. It was showed that this assumption did not have an impact on the results (see Fig. 2).
- The only clusters explicitly computed are $\mathrm{He}_{x \le 6}$ (i.e. $N=6$) whereas Faney’s work explicitly accounted for clusters up to $\mathrm{V}_{50}\mathrm{He}_{250}$ and solved a bigger system of equations. The influence of this threshold *N* above which clusters are integrated in the quantity $c_b$ is discussed in section "Influence of N".
- Clusters containing more than six He atoms are assumed to be immobile (i.e. $D_i = 0$ for $i>6$) due to trap mutation events. This assumption is motivated by DFT and MD results suggesting that the self-trapping energy is below the binding energy of one He atom in a pure He cluster for clusters containing more than five He atoms^3^.
  For smaller clusters ($\mathrm{He}_1$, $\mathrm{He}_2$, ..., $\mathrm{He}_6$) the diffusion coefficient and the dissociation by He emission energy vary with the number of He atoms in the cluster (see Table 1).

**Table 1:** Pure He clusters properties in W. Diffusion properties are taken from Faney *et al.*^30^ and binding energies are taken from Becquart *et al.*^36^.

Cluster	$D_0\, ({\text{m}^{2} \,\text{s}^{-1}})$	$E_{\mathrm{diff}} \, (\text{eV})$	$E_b \, (\text{eV})$
He$_1$	$2.95\times 10^{-8}$	0.13	–
He$_2$	$3.24\times 10^{-8}$	0.20	1.0
He$_3$	$2.26\times 10^{-8}$	0.25	1.5
He$_4$	$1.68\times 10^{-8}$	0.20	1.5
He$_5$	$5.20\times 10^{-9}$	0.12	1.6
He$_6$	$1.20\times 10^{-9}$	0.30	2.0

This He transport model was implemented in Python and solved using the finite element solving platform FEniCS^37^. All plots in this work were generated with Matplotlib^38^.

## Results

In this section, the current implementation is first compared with the one from Faney^30^ to ensure the additional assumptions do not produce different results. A standard half-slab case is then described and a parametric study is performed by varying the exposure conditions. Finally, the model is compared against experimental data.

### Tendril case

The tendril application case described in^30^ was simulated in 1D with the current implementation and the results were compared.

The domain size is 30 nm and the volumetric source term is described as follows:

14 $$\begin{aligned} \Gamma (x) = \varphi _{\mathrm{imp}} \, f(x) \end{aligned}$$

where $\varphi _{\mathrm{imp}} = {5 \times 10^{25}}\,{\text{m}^{-2} \,\text{s}^{-1}}$ is the implanted He flux and *f*(*x*) is a Gaussian distribution with a mean value $\mu = R_p = {1.5}\,\text{nm}$ and a standard deviation $\sigma = {1}\,\text{nm}$ which corresponds to a 100 eV He implantation based on SRIM computations^39^.

Mobile He clusters concentrations were set to zero at the tendril’s surfaces ($x={0}\,\text{nm}$ and $x={30}\,\text{nm}$).

Concentration profiles computed by the current implementation showed good agreement with the ones obtained by Faney et al.^30^ (see Fig. 2). The discrepancies are likely due to a difference in the set of dissociation energies that have been used. Indeed, at low temperature, where dissociation is not activated, the discrepancies were very small whereas at high temperature, differences increased because dissociation became more dominant. When $c_b$ is small compared to $c_{\mathrm{He}_1}$, the equilibrium of $\mathrm{He}_1$ is independent of these dissociation energies and the profiles for $\mathrm{He}_1$ are identical.

Moreover, increasing the temperature tended to inhibit bubble formation in the tendril. This was explained by a greater increase in the dissociation rate and in losses at surfaces than the increase in the clustering rate. This observation is in agreement with MD results simulating He implantation in tendrils^40,41^. The current implementation and the additional assumptions that were made are therefore valid.

**Figure 3:**
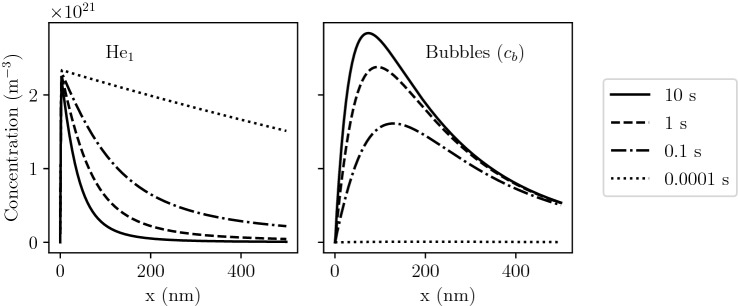
Concentration profiles of He$_1$ (left) and bubbles (right) in W exposed to 100 eV He at $10^{22}\,{\text{m}^{-2} \,\text{s}^{-1}}$ and 1000 K.

### Half-slab case

He transport was simulated in a 1D semi-infinite W slab. This case is the standard case describing the main quantities of interest of the parametric study performed in section "Influence of N".

The domain size is 0.1 mm which is much greater than the penetration depth of He in the simulations. 100 eV He were implanted in the first 1.5 nm as in section "Tendril case". The implanted flux was $1 \times 10^{22}\,{\text{m}^{-2}\, \text{s}^{-1}}$ and the temperature was 1000 K.

At low fluences, He diffused really quickly into the bulk (see Fig. 3) and the bubbles concentration $c_b$ was found to be zero. As the fluence increased, bubbles started to appear and acted as strong sinks for mobile He. This lead to a great decrease in the mobile He concentration profile.

It is worth noticing the maximum of $c_b$ was not located at the maximum of $c_{\mathrm{He}_1}$ which is the implantation depth $R_p$. This was explained by the diffusion of small mobile clusters as shown by analytical models^42^. As He clusters, small mobile clusters diffuse deeper into the bulk until trap-mutation occurs and bubbles nucleons (clusters with more than 6 He) are created. From that point, bubbles are formed relatively far from the surface. Because He is implanted in the first nanometres, $c_{\mathrm{He}_1}$ is maximum at $R_p = {1.5}$nm and interactions with bubbles are stronger in this region. This tends to draw the maximum location of $c_b$ towards the surface.

**Figure 4:**
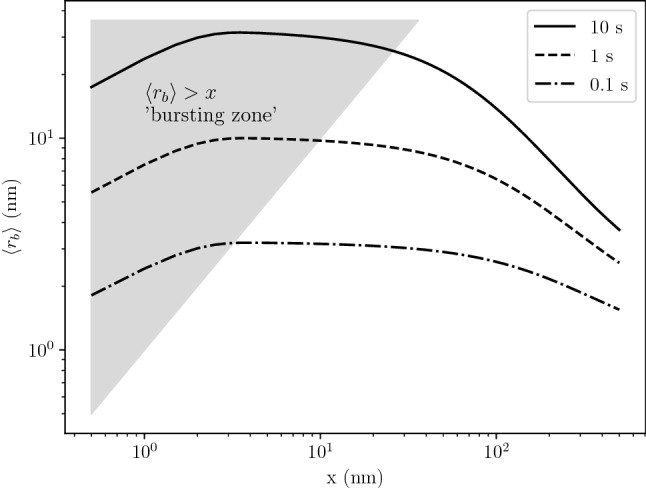
Profile of mean bubble radius $\langle r_b \rangle$ as a function of depth *x* in W exposed to 100 eV He at $10^{22}\,{\text{m}^{-2} \,\text{s}^{-1}}$ and 1000 K.

The He content in bubbles $\langle i_b \rangle$ and the radius $\langle r_b \rangle$ were computed. After 10 s of implantation, bubbles located in the near surface contained up to $3 \times 10^{7}$ He. The maximum of $\langle r_b \rangle$ was found to be very close to the surface at approximately 2 nm (see Fig. 4). This is explained by the high concentration of mobile He in this near surface region. Moreover, a bursting zone can be defined by the region where $\langle r_b \rangle$ is greater than the depth of the bubble. In this region, bubble of this size would have likely burst.

**Figure 5:**
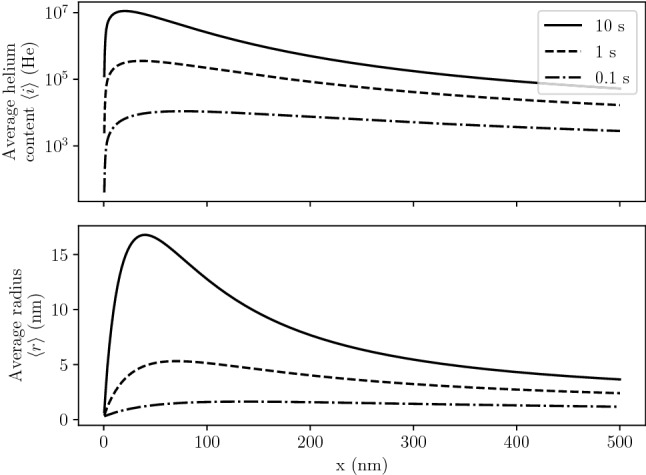
Average helium content $\langle i \rangle$ and average radius $\langle r \rangle$ in all clusters (mobile and bubbles) in W exposed to 100 eV He at $10^{22}\,{\text{m}^{-2}\, \text{s}^{-1}}$ and 1000 K.

From this average He content in bubbles and from Equations 13 and 12 expressing the clusters radii, the average radius $\langle r \rangle$ can be computed as:

15 $$\begin{aligned} \langle r \rangle = \frac{\sum _{i=1}^\infty c_i r_i}{\sum _{i=1}^\infty c_i} = \frac{\sum _{i=1}^N c_i r_i + c_b \langle r_b \rangle }{\sum _{i=1}^N c_i + c_b} \end{aligned}$$

The average content of He in all clusters $\langle i \rangle$ is computed in a similar way:

16 $$\begin{aligned} \langle i \rangle = \frac{\sum _{i=1}^\infty c_i i}{\sum _{i=1}^\infty c_i} = \frac{\sum _{i=1}^N c_i i + c_b \langle i_b \rangle }{\sum _{i=1}^N c_i + c_b} \end{aligned}$$

These two quantities are comparable to the ones obtained by Faney *et al.*^30^. After 100 s of exposure, the average radius 50 nm below the surface was above 10 nm (see Fig. 5). Moreover, the location of the maximum of these quantities move towards the exposed surface.

The average radius $\langle r \rangle$ cannot be easily compared to experimental observations for it includes contributions from very small mobile He$_x$ clusters which are not visible experimentally (only bubbles with a radius greater than 1–3 nm are observable depending on the observation technique).

### Influence of exposure parameters on He bubble growth

The impact of He flux and temperature *T* was studied on the case described in section "Half-slab case" in order to identify trends. Behaviour laws are identified and can be used to obtain information on He transport without needing to run any simulation.

#### Parametric study

A parametric study was performed by varying the implanted flux $\varphi _{\mathrm{imp}}$ between ${1 \times 10^{17}}\,{\text{m}^{-2} \,\text{s}^{-1}}$ and ${5 \times 10^{21}}\,{\text{m}^{-2} \,\text{s}^{-1}}$ and the sample temperature *T* between 100 K and 1200 K.

Several quantities were computed. First the bubbles inventory is defined as:

17 $$\begin{aligned} I_{\mathrm{bubbles}}= \displaystyle \int c_b \, dx \end{aligned}$$

The total helium inventory is calculated by:

18 $$\begin{aligned} I = \displaystyle \int \sum _{i=1}^N i c_i + \langle i_b \rangle c_b \, dx \approx \displaystyle \int \langle i_b \rangle c_b \, dx \end{aligned}$$

The spatial mean helium content in bubbles can be computed as:

19 $$\begin{aligned} {\langle {\bar{i}_{b}} \rangle } = \frac{\displaystyle \int \langle i_b \rangle c_b \, dx}{\displaystyle \int c_b \, dx} \approx \frac{I}{I_{\mathrm{bubbles}}} \end{aligned}$$

The approximation made in Equations 18 and 19 is valid as long as $\int \langle i_b \rangle c_b dx \gg \int \sum _{i=1}^N i c_i dx$ (i.e. the He inventory is dominated by that of the bubbles). This is the case in these simulations because $N=6$ (the influence of this parameter is discussed in section "Influence of N").

**Figure 6:**
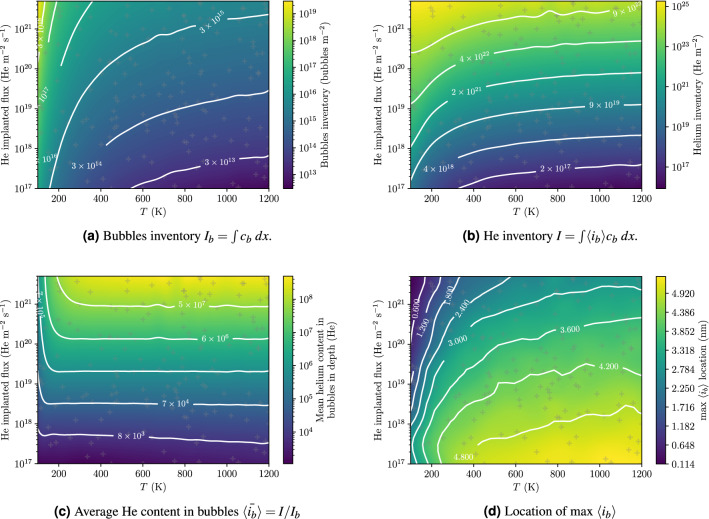
Evolution of quantities as a function of the implanted flux and temperature after 1 h of 100 eV He exposure. Grey crosses correspond to simulations points.

**Figure 7:**
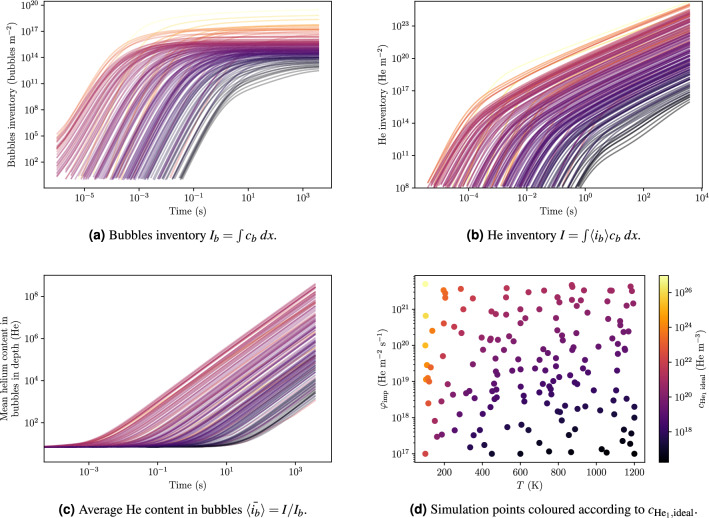
Temporal evolution of quantities in W exposed to 100 eV He at $10^{22}\,{\text{m}^{-2}\, \text{s}^{-1}}$ and 1000 K for temperatures varying from 120 K to 1200 K and implanted fluxes varying from ${10^{17}}\,{\text{m}^{-2} \,\text{s}^{-1}}$ to $10^{21}\,{\text{m}^{-2}\, \text{s}^{-1}}$. Each line corresponds to a simulation point (grey crosses on Fig. 6a and points on **d**). The lines are coloured according to the parameter $c_{\mathrm{He}_1, {\mathrm{ideal}}} = \varphi _{\mathrm{imp}} \, R_p/D(T)$ with $R_p = {1.5}\,\text{nm}$ and *D* the diffusion coefficient of $\mathrm{He}_1$ in W.

More than 160 simulations were performed simulating 1 h of exposure. For each simulation, the quantities of interest described above were computed. A Gaussian regression process^43^ was used to interpolate the data based on Bayesian inference as done in^33^ (see Fig. 6). The temporal evolution of these quantities was also assessed (see Fig. 7).

After 1 h of exposure, the bubbles inventory $I_{\mathrm{bubbles}}$ shows a weak dependence on temperature at high temperature and a weak dependence on the implanted flux at low temperature (see Fig. 6a). $I_{\mathrm{bubbles}}$ varies from ${4 \times 10^{12}}{\text{bubbles } \,m^{-2}}$ at high temperature and low flux to ${2 \times 10^{19}}{\text{bubbles } m^{-2}}$ at low temperature and high flux.

The He inventory *I* varies from ${8 \times 10^{16}}\,{\text{m}^{-2}}$ at high temperature and low flux to${10^{25}}\,{\text{m}^{-2}}$ at low temperature and high flux (see Fig. 6b). For temperatures above 600 K, the temperature dependence is rather weak compared to the flux dependence.

For temperatures above 300 K, and after 1 h of exposure, the sample temperature does not impact the value of ${\langle {\bar{i}_{b}} \rangle }$ (see Fig. 6c). The mean He content increases with the implanted flux as expected and varies between $10^{3}$He at low flux and ${5 \times 10^{8}}$He at high flux.

The position of the maximum of $\langle i_b \rangle$ tended to increase with temperature and decrease with implanted flux (see Fig. 6d). After 1 h of exposure, it was found to be really close to the surface down to 0.1 nm at low temperatures and high fluxes. The validity of the model in this region of the parameter space is questionable considering that the bubble radius is greater that the thickness of the ligament between the edge of the bubble and the surface. Such a bubble would therefore have burst before reaching this size.

For each simulation point, the temporal evolution of the quantities described above has been computed. To better identify the time series on the $\varphi _{\mathrm{imp}}, T$ plane, lines have been coloured according to the parameter $c_{\mathrm{He}_1, \mathrm{ideal}}$ which is a function of both the implanted flux and the temperature (see Equation 20) expressed in m $^{-3}$.

20 $$\begin{aligned} c_{\mathrm{He}_1, \mathrm{ideal}} = \frac{\varphi _{\mathrm{imp}} \, R_p}{D(T)} \end{aligned}$$

where $\varphi _{\mathrm{imp}}$ is the implanted flux, *D* is the diffusion coefficient of mobile $\mathrm{He}_1$ in W (see Table 1), $R_p = {1.5}\,\text{nm}$ is the implantation depth and *T* is the temperature in K.

All these quantities showed a similar behaviour in time even though the kinetics were found to be different (see Fig. 7). For instance, for each $(T, \varphi _{\mathrm{imp}})$ couple, $I_{\mathrm{bubbles}}$ first increased as a power law of time before reaching a maximum (see Fig. 7a). The total He inventory *I* increased with time and for each simulation point but the growth rate decreased at long exposure times (see Fig. 7b). This phenomenon is explained in details in section "Influence of N". Similarly, ${\langle {\bar{i}_{b}} \rangle }$ could be written as a power law of time described in Eq 21 (see Fig. 7c). The depth of the maximum of $\langle i_b \rangle$ tended to decrease with time as it was observed in section "Half-slab case" (see Fig. 7a).

**Figure 8:**
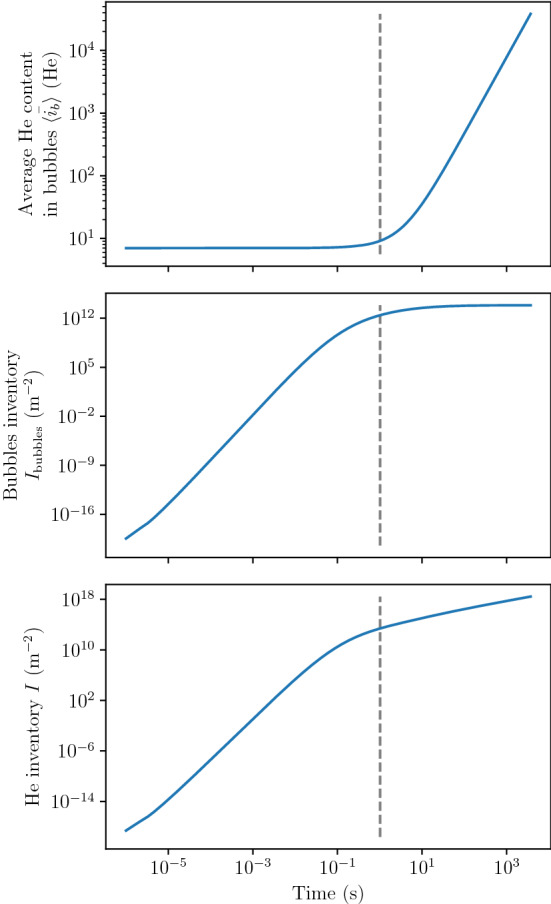
Temporal evolution of ${\langle {\bar{i}_{b}} \rangle }$, $I_{\mathrm{bubbles}}$ and *I* in W exposed to 100 eV He at ${1.59 \times 10^{18}}\,{\text{m}^{-2} \,\text{s}^{-1}}$ and 1020 K. The dashed grey vertical line represents the transition from nucleation regime to bubble growth regime.

#### Identification of regimes for inventory evolution

For every $(T, \varphi _{\mathrm{imp}})$ couple, $I_{\mathrm{bubbles}}$ increased rapidly at low fluences until reaching a maximum at high fluences (see Fig. 7a). On the other hand, the mean He content ${\langle {\bar{i}_{b}} \rangle }$ was constant at low fluences and increased as a power law of time at high fluences (see Fig. 7c). The evolution of ${\langle {\bar{i}_{b}} \rangle }$ can be described as:

21 $$\begin{aligned} {\langle {\bar{i}_{b}} \rangle } = N + 1 + a \, t^b \end{aligned}$$

where $N=6$ in this model, *a* and *b* depend on $(T, \varphi _{\mathrm{imp}})$. The choice of $N=6$ in this model is detailed in section "Influence of N". The total He inventory *I* being the product of these two quantities, two different growth rates were observed (see Figs. 7b and 8).

This phenomenon can be attributed to two different regimes. The first regime is the nucleation regime where new bubbles nucleons are created (i.e. $c_b$ and $I_{\mathrm{bubbles}}$ increase). In the nucleation regime, the bubble concentration $c_b$ and the capture radius $\langle r_b \rangle$ are too low for the He content in bubbles $\langle i_b \rangle$ to increase significantly (i.e. ${\langle {\bar{i}_{b}} \rangle }$ is constant). The second regime is the bubble growth regime. In this regime, $c_b$ is high enough for interactions between bubbles and mobile He to occur. Implanted interstitial He atoms ($c_{\mathrm{He}_1}$) therefore interact preferably with bubbles rather than clustering with other interstitial He atoms. This means that no additional bubbles nucleons are created (i.e. $c_b$ reaches a maximum). Because interactions between bubbles and mobile He are strong, the term $\langle k_b^+ \rangle c_1 c_b$ in Equation 11 becomes significant and the He content increases (i.e. ${\langle {\bar{i}_{b}} \rangle }$ increases). This is illustrated by the thickness of the interaction arrows in Fig. 1.

#### Influence of *N*

In order to assess the impact of the parameter *N* in Equation 11, the evolution of the He inventory *I*, the mean He content in immobile clusters (different from ${\langle {\bar{i}_{b}} \rangle }$) and the bubbles inventory $I_{\mathrm{bubbles}}$ was computed with several values of *N*.

The flux of 100 eV He in this test case was $10^{20}\,{\text{m}^{-2} \,\text{s}^{-1}}$ and the temperature was 1000 K.

**Figure 9:**
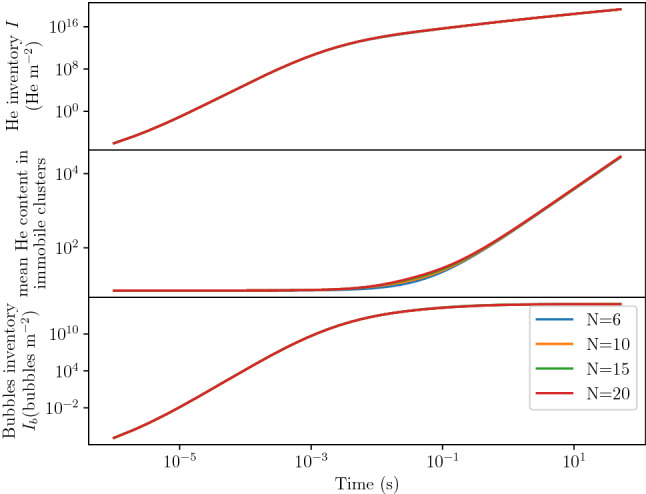
Comparison of several quantities of interest for several values of *N* in W exposed to 100 eV He at $10^{20}\,{\text{m}^{-2}\, \text{s}^{-1}}$ and 1000 K.

It was shown that varying *N* had no impact on these quantities whatsoever (see Fig. 9). This highlights the very quick transition from nucleation regime to growth regime in this model.

The number of equations that need to be solved can therefore be minimised by setting the parameter *N* to its minimum ($N=6$) without losing accuracy in the results. This minimum value corresponds to the number of mobile clusters which have to be explicitly simulated in order to account for all the diffusion mechanisms.

**Figure 10:**
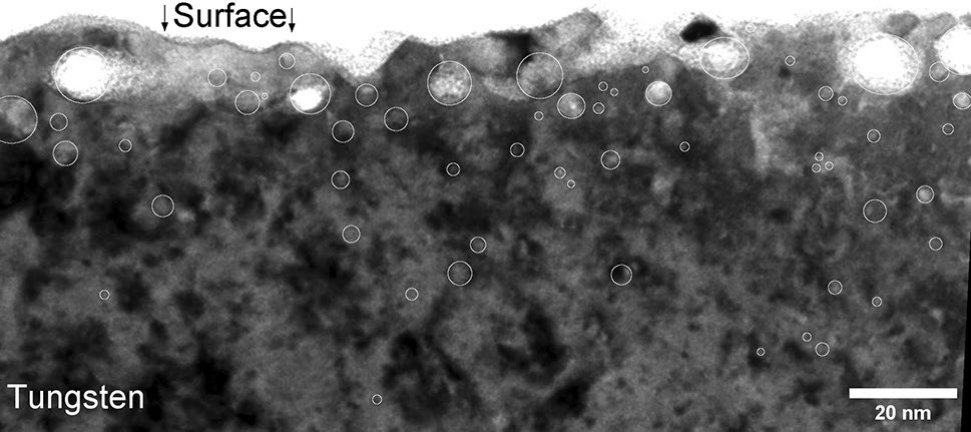
TEM images of W after exposure to 75 eV He at ${2.3 \times 10^{22}}\,{\text{m}^{-2}\,\text{s}^{-1}}$ and 1053 K for 13 s showing bubbles that have burst, large size bubbles at the near surface and small size bubbles in the bulk.

**Figure 11:**
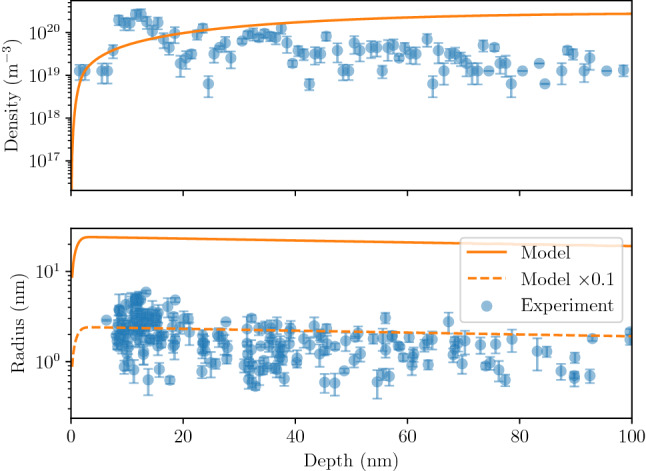
Comparison of experimental results with simulations for W implanted with 75 eV He at ${2.3 \times 10^{22}}\,{\text{m}^{-2}\, \text{s}^{-1}}$ and 1053 K for 13 s. Error bars correspond to the lowest and highest radius in the TEM image.

### Comparison with experiments

He implantation experiments were performed on W in the linear plasma device PSI–2 ^44^. W was irradiated with 75 eV He at ${2.3 \times 10^{22}}\,{\text{m}^{-2}\, \text{s}^{-1}}$ and 1053 K for 13 s. A thin lamella for cross-sectional observations was prepared using the FIB (Focused Ion Beam) technique with a Dual Beam FIB (FEI Helios 600 NanoLab). Prior to FIB cutting, the surface of the sample was coated with a SiO layer for better contrast and then with a protective platinum layer to avoid damaging the surface during the lamella preparation. Cross-sectional observations of the He-implanted W were performed using transmission electron microscopy in a TEM FEI Titan 80-300 apparatus. A typical TEM image of the lamella is presented in Fig. 10. Comparison of under- and over-focused TEM images allowed identification of the bubbles. Bubbles were observed up to 100 nm with larger bubbles closer to the surface and smaller bubbles deeper in the bulk. Open bubbles and holes at the surface were also observed suggesting bursting events occurred. This is in accordance with what was observed in the simulations (see Fig. 4).

A procedure was developed to automate the bubble detection on TEM images using the ImageJ software ^45^. The area of bubbles were computed as well as their diameter assuming a spherical shape for the bubbles. Bubble density and size as a function of depth was therefore computed using 12 pairs of under- and over-focused TEM images. The bubble density was found to range from ${7 \times 10^{19}}\,{\text{m}^{-3}}$ to ${2 \times 10^{20}}\,{\text{m}^{-3}}$ and the bubble radius ranged between 1 nm and 10 nm (see Fig. 11). Although the resolution of the TEM is below 1 nm, the number of bubbles with radius below 2 nm is underestimated due to the limited contrast.

This experiment was simulated using the same exposure conditions. The simulated bubbles density $c_b$ was found to be in accordance with the one measured experimentally. Some discrepancies were found at the near surface.

The bubble radius ($\langle r_b \rangle$) is however overestimated by an order of magnitude compared to experimental measurements. This could imply that the current model linking the He content to the bubble radius is overestimated and that a more accurate one is needed. Finally, it would be worth investigating this further to determine if some saturation in the bubble size occurs and the impact of initial defects.

## Conclusion

A simplified model for He clustering in W based on the existing model by Faney *et al.*^29,30^ was presented. The set of coupled equations of this model was solved using the finite element method. Medium sized clusters were not explicitly accounted for and all immobile clusters were described by the grouped quantity $c_b$. Interactions with self interstitial W atoms produced by trap mutation events were neglected. Similarly, dissociations by vacancy emission have been assumed to be negligible compared to dissociations by He emission.

This model was compared to existing numerical results. It was found that the results produced by the current simplified model were very similar. This allows to identify non-dominant processes that can be safely neglected. Moreover, reducing the number of equations (only eight in this model compared to thousands in Faney’s work) greatly reduces the computational cost of the solver. As an example, one simulation point in section "Influence of N" took only a few minutes to run on a non-parallelised solver.

This novel simplified implementation was then applied to a semi-infinite W slab exposed to 100 eV He. The implanted flux was $10^{22}\,{\text{m}^{-2} \,\text{s}^{-1}}$ and the exposure time was 10 s. The maximum bubble size was located in the first few nanometres below the surface which was in very good agreement with experimental observations. The maximum average radius was 15 nm located around 50 nm below the surface.

A parametric study was performed to investigate the influence of both He implantation flux and temperature. Several quantities of interest were computed such as bubbles inventory, total He inventory or average He content in bubbles. Two distinct growth regimes were found namely the nucleation regime in which new bubbles are created and the growth regime in which existing bubbles increase in size.

Comparison with experimental measurements was performed. The bubble density predicted by the model was found to be in good accordance with experimental observations. The predicted bubble radius was however overestimated. It suggests that the model linking He content to cluster radius may need to be adapted for large bubbles.

This model however has a few caveats, some of which have already been discussed by Faney *et al.*^30^. First, the He-to-vacancy ratio of 4:1 in this work differs from MD studies^35,46^. The dilute limit approximation may also not be valid as soon a the volume fraction occupied by He becomes comparable to unity. The volume fraction of He is defined by $V = \sum 4/3\pi r_i^3 c_i$. The diffusion limited reaction model may therefore not be valid for very large bubbles and/or very high concentrations. Then, as observable on Fig. 7c, the He content in bubbles $\langle i_b \rangle$ keeps increasing with time. One could argue that some physical processes would tend to limit or even avoid this growth at some point. This is particularly true for bubbles very close to the surface that would tend to burst and release their He content to the atmosphere. Implementing a bursting model similar to the one developed by Blondel *et al.*^31^ could help alleviate this issue. He diffusion may also be limited by mechanical stress induced by pressurised bubbles and He presence in the material, suggesting the current model gives only an upper estimation of the He clusters size in the sample. Similarly, this model does not account for bubbles coalescence which would tend to decrease bubbles concentrations while increasing their sizes. Finally, the approximation made by $\langle r_b \rangle \approx f(\langle i_b \rangle )$ is valid when the bubble size distribution does not deviate much from $\langle i_b \rangle$. Faney et al. suggested that this is the case for large bubbles^30^.

In future work, this model will be used to estimate bubble production induced by indirect He production (tritium decay and W transmutation). The use of more accurate correlations for He bubbles growth^35,46^ would tend to reduce the overall He content. Additional bubble models such as bursting or coalescence will also be investigated.
